# β-Amyloid (Aβ) and Human Cathelicidin LL-37: Two Sides of the Same Coin?

**DOI:** 10.3390/ijms27125460

**Published:** 2026-06-17

**Authors:** Anna Lia Asti

**Affiliations:** Department of Medical and Surgical Sciences, Alma Mater Studiorum, Università di Bologna, 40126 Bologna, Italy; annalia.asti@unipv.it

**Keywords:** β-amyloid (Aβ), hCAP18 cathelicidin (LL-37), antimicrobial peptide (AMP), lipopolysaccharide (LPS), aggregation-prone regions (ARPs), critical micelle concentration (CMC), Alzheimer’s disease (AD), Parkinson’s disease (PD)

## Abstract

Physiologically produced circulating β-amyloid (Aβ) exerts critical physiological functions. Although Aβ is a key player in Alzheimer’s disease (AD), it may initially be beneficial at the onset of infection. As an evolutionary conserved antimicrobial peptide (AMP), Aβ contributes to innate immune defense against pathogens. Host defense peptides such as Aβ and human cathelicidin (LL-37) not only kill pathogens through their antimicrobial activity but also exhibit high affinity for bacterial lipopolysaccharides (LPSs) and membrane receptors. LL-37, which is upregulated in the brain, binds to Aβ, modulating its aggregation; Aβ and LL-37 are protective under physiological conditions, but during chronic infection or dysregulation, their interaction becomes toxic and contributes to AD pathology. Similarly to Aβ, LL-37 can induce neuroinflammation by stimulating human microglia to release inflammatory cytokines, such as TNF-α and IL-6. Neuroinflammation is essential for protecting the brain from pathogens—when prolonged, it drives pathological processes underlying AD, Parkinson’s disease (PD), and other neurodegenerative disorders.

## 1. Introduction

AD is a neurodegenerative disease whose causes remain unclear, preventing the development of effective therapies. Significant progress has been made in studying the role of neuroinflammation, leading to the development of new therapies that can suppress the inflammatory response. However, the identification of new therapeutic compounds that can enhance the innate immune system while inhibiting Aβ aggregation remains a major challenge [1].

Aβ is a key player in the pathogenesis of amyloidosis [2,3] and AD [4], exhibiting two common motifs: cationic charge and amphiphilic structure. The amphiphilic nature of AMPs [5,6] enables them to penetrate lipid membranes. The spontaneous aggregation of proteins into ordered structures is a fundamental process; protein–membrane interactions can induce conformational changes that favor intermolecular connections, leading to aggregation [1,5]. The extent to which Aβ interacts with lipid membranes depends on its aggregation state.

LL-37 is also an amphipathic molecule and a host defense peptide [7] involved in immune protection. LL-37, released by neutrophil degranulation or secreted by other cell types during infection, modulates the innate immune system [6,7] and inflammatory response which is one of the main components of AD progression [8]. LL-37 is upregulated in the brain, where it binds to Aβ, and modulates its aggregation. Both Aβ and LL-37 exert protective functions initially, but under chronic infection or dysregulation their interaction becomes toxic [7]. These peptides not only kill pathogens through their antimicrobial activity but also exhibit high affinity for bacterial LPSs and membrane receptors. The interaction between LL-37 and LPS can lead to the formation of stable peptide–lipid complexes, including amyloid-like fibers [8]. The rationale for investigating the Aβ–LL-37 axis lies in the limited therapeutic options currently available for AD, and the promising properties of AMPs. The considerations presented in this paper aim to support the search for reagents or drugs capable of modulating Aβ and LL-37 in neurodegenerative disease.

## 2. Structure and Function of Aβ Peptide

Aβ_1–42_ is a 42 amino acid peptide produced from the amyloid precursor protein (APP) though two subsequential proteolytic cleavages by β-secretase (BACE1) and γ-secretase at intramembranous sites [9,10]. Aβ is generated within acidic intracellular compartments such as the early endosome or Golgi complex [11]. Among Aβ isoforms, Aβ_1–42_ is considered more neurotoxic than Aβ_1–40_ and is the primary component of amyloid plaques in the brain.

**Figure 1:**
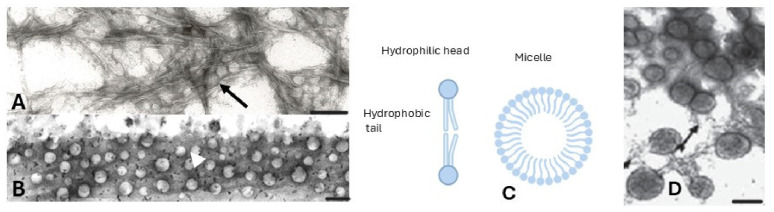
Transmission electron microscopy of Aβ and LPS. (**A**) Long Aβ fibrils with regular helical twists are detectable. Little micelles are widely spread among them (arrow). Bar = 100 nm. (**B**) Bacterial LPS displays surfactant properties, and it may aggregate into different physical structures such as micelles (arrowhead). Bar = 200 nm. (**C**) Micelle structure. (**D**) Micelles are formed by concomitant action of Aβ and LPS (hetero micelles). This indicates a direct affinity at molecular level. Some older micellar particles are releasing aggregate from the surface (arrow). Bar = 200 nm [21].

Aβ fibril with a diameter of 7–12 nm assembles into β-sheet-rich fibrils, as observed by X-ray diffraction, and constitutes the main component of amyloid plaques. Their stability arises from the β-sheet conformation [8] and extensive hydrogen bonding. Aggregation kinetics are influenced by factors such as pH, ionic strength, and temperature. Under appropriate conditions, every protein can aggregate into amyloids [10]. A switchable sequence present in both aggregated and soluble state may allow transition, depending on environmental conditions and concentration [11]. Aβ_42_, considered the more neurotoxic species and the primary component of amyloid plaques in the brain, is two amino acids longer than Aβ_1–40_, and Aβ_42_/Aβ_40_ is a critical factor in AD progression. Multiple physiological roles have been proposed for Aβ, including regulation of cholesterol transport, protection against oxidative stress, and activation of key kinases [12]. In this work, Aβ_1–42_ peptide was studied for antimicrobial activity. As an AMP-like molecule, Aβ_1–42_ [5] may help contain invading pathogens, and its production might initially be beneficial at the onset of infection. According to the antimicrobial protection hypothesis, Aβ functions as an AMP within the brain’s innate immune system [5]. Soluble Aβ oligomers bind to carbohydrates on the surface of invading microbes (bacteria, viruses, or fungi) via heparin-binding domain [13]. Aβ aggregation proceeds through nucleation of free disordered monomers [14,15]. The process begins with the formation of micelle-like assemblies (Figure 1A), followed by the rearrangement of monomers into stable ordered nuclei. Aggregation kinetics are concentration-dependent. Insoluble plaques may be less toxic than smaller, more diffusible oligomers. Oligomerization in particular is viewed as a pathogenic pathway and Aβ oligomers are assumed to be intrinsically abnormal, but oligomerization and fibrillization appear to mediate Aβ’s protective activity [15].

According to the model proposed by Lomakin et al. [16], Aβ fibrillogenesis is influenced by a certain critical micellar concentration (CMC). Fibrils can nucleate within these micelles (Figure 1A) or on heterogeneous nuclei (seeds) (Figure 1D), consistent with reported Aβ surfactant properties. Aβ concentration in plasma or cerebrospinal fluid is very low [16,17]; homogeneous fibril nucleation is unlikely to occur. The kinetics of aggregation might also be enhanced through interaction with apolipoprotein E4 or other molecules [18]. Factors known to affect CMC in aqueous solutions include surfactant structure, electrolyte concentration, counterions type, the presence of organic solutes, and temperature [19]. Studies have shown that the aggregate structure can be modulated by varying the length of the hydrophobic tail, the flexible linker, or both [20].

If Aβ acts through an antimicrobial mechanism, fibrils may represent a response of the innate immune system that correctly or incorrectly perceives an infection [3]. It remains unclear whether Aβ-mediated activation of immune pathways in AD reflects pathological dysregulation or an immune response to chronic infection [22,23]. The observation of low Aβ_42_ levels in patients with tuberculous meningitis (TBM) [24] should be considered in light of the recent evidence suggesting a possible antimicrobial role for Aβ [6,24] and a hypothetical infectious “trigger” for AD.

AMPs, like antibiotic agents, require a minimum inhibitory concentration (MIC) to prevent bacterial growth, and can be bacteriostatic or bactericidal depending on peptide concentration [25]. Aβ oligomers, through direct interaction with isolated mitochondria or indirect association with neuronal membranes, induce intracellular calcium deregulation that triggers apoptosis via mitochondrial dysfunction [26]. Low AMP levels can severely compromise immunity and increase barrier permeability, allowing inflammatory mediators or pathogens to reach the central nervous system and activate microglia.

Neurodegeneration induced by soluble inflammatory mediators is not the only mechanism by which microglia contribute to neuronal death. Recurrent reactivation of chronic latent infections [21] stimulates Aβ production, progressively leading to impaired clearance, neurodegeneration, and neuroinflammation [27]. Over time, chronic neuroinflammation produces distinct alterations in the brain that contribute to neuronal degeneration, and consequently, to functional decline [28]. Aβ deposits activate microglia that express immunological receptors, such as TLR2, TLR4, and TLR6, as well as their co-receptors, including CD36, CD14, and CD47 [29], which can act together, triggering the microglial response to Aβ [30]. Persistent Aβ aggregation prevents the resolution of inflammation and results in a chronic inflammatory state [31]. This contrasts with the normal immune response to pathogens which subside once the infection is cleared [32]. Impaired degradation of Aβ has been associated with reduced lysosomal and endosomal enzyme activity in patients with AD [33,34]. A chronic state of inflammation, termed “inflammaging”, increases gut and BBB permeability, influencing Aβ production in the brain [35,36]. Inflammasome activation, particularly via NLRP3, amplifies neuroinflammatory signaling [36] and regulates caspase activation. Aβ-mediated stimulation induces neurodegeneration through soluble inflammatory factors, and increases the exposure of phosphatidylserine on neuronal processes, enhancing microglia uptake. This susceptibility depends on the activation state of the innate immune system [37].

### Role of Aggregation-Prone Regions (APRs) in Amyloidogenic Peptides

Protein aggregation is driven by short specific segments composed of 5–15 residues, known as APRs [38]. APRs are mainly hydrophobic and are typically buried within the core of the folded protein; if the protein is unfolded, the APRs become exposed and trigger aggregation [39]. Amyloidogenic peptides derived from APRs can co-aggregate and interfere with the function of other proteins that share similar APR sequences [38].

APRs are crucial for the interaction with LL-37 because these specific 5–15 amino acid residues constitute the Aβ core for fibril formation. LL-37 binds to these regions and modulates their self-association, a key mechanism by which it inhibits the formation of toxic β-sheet-rich amyloid plaques in the brain [39].

Proteins have evolved to optimize their aggregation risk in cellular environments by minimizing aggregation-prone regions while preserving those essential for proper folding and function [40]. Synthetic amyloid peptides designed from APRs have been widely explored for therapeutic medical applications [41]. APRs can promote structural order, stabilize proteins in their native states, and serve as sites for protein–protein interactions [42], including antibody–antigen interfaces [43,44].

## 3. The Biological Function of LL-37, a Host-Defense Peptide

**Figure 2:**
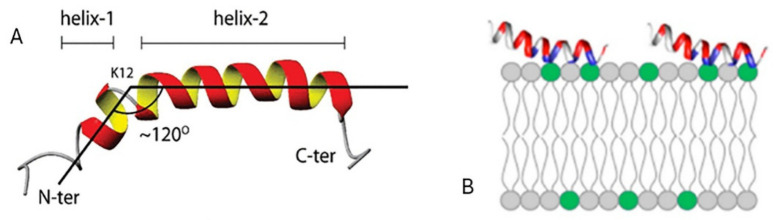
NMR structure of LL-37 in dodecylphosphocholine micelles (**A**) [53] showing the angle between the two helical domains and the break point centered at K12; (**B**) LL-37-forming pore on bacterial membrane.

hCAP-18 is the only human cathelicidin, also known as LL-37 [45] (Figure 2A). It is expressed or synthesized in response to bacterial stimuli [46,47] or their products, and it is an important effector molecule of the innate immune system [48].

Structurally, LL-37 is a 37 amino acid peptide with two leucines at the N-terminus. Its distribution of 16 charged residues, combined with both hydrophilic and hydrophobic functional groups, gives the peptide amphipathic properties.

As an AMP, LL-37 exhibits two features common to many AMPs: cationic charge and amphiphilic form, which facilitates membrane disruption; LL-37 tends to inhibit the formation of Aβ fibrils [49] because it binds more strongly to Aβ oligomers than to fibrillar forms. In the presence of a phospholipid bilayer, LL-37 adopts an α-helical conformation [49]; its direct interaction is mainly involved in the disintegration of the microbial cell wall (Figure 2B), leading to cell death. High levels of LL-37 are found in the gastrointestinal tract and brain [50]. Several endogenous factors, including inflammatory cytokines, growth factors, and the active form of vitamin D can induce LL-37 expression [51]. Low LL-37 levels are associated with an increased risk of severe infections, whereas elevated levels of LL-37 concentrations have been linked to the pathology of several non-infectious diseases, such as atherosclerotic plaques [52].

Insufficient levels of Vitamin D3 can lead to the under-expression of LL-37, reducing the host’s ability to counteract infections [54]. Vitamin D acts through the vitamin D receptor (VDR), which directly regulates the transcription of the CAMP gene encoding LL-37. The biological relevance of LL-37 is further highlighted by its specific interaction with the severe acute respiratory syndrome SARS-CoV2 spike proteins S1, which, due to the obstruction of S1’s receptor-binding domain, inhibits host cell binding and can also interact with the ligand-binding domain of ACE2, the receptor-mediating SARS-CoV2 viral entry [55]. In animal studies, intraperitoneal administration of the murine cathelicidin peptide CRAMP would appear to protect non-obese diabetic (NOD) mice from developing autoimmune diabetes [56].

LL-37 also plays an important role in autoimmune responses. In systemic lupus erythematosus (SLE) and psoriasis, LL-37 forms complexes with self-nucleic acids (DNA/RNA) that break immunological tolerance, trigger inflammation, and drive autoimmune activation [57]. LL-37-nucleic acid complexes are potent stimulators of plasmacytoid dendritic cells (pDCs), which serve as a bridge between innate and adaptative immunity leading to type 1 INF production [38]. In addition, LL-37 can directly activate mast cells, inducing the production of cytokines such as IL-1β, IL-2,4,5,6, TNF, GM, CSF, and various chemokines [58].

### 3.1. Central Role of LL-37 in Neutrophil Extracellular Trap (NET) Formation

Within neutrophils, hCAP-18 is stored as an inactive precursor in peroxidase-negative granules [59,60]. During the process of NETosis (neutrophil extracellular trap formation), hCAP-18 is cleaved into its active form. LL-37 becomes tightly associated with nuclear chromatin fibers, a process that reduces its antimicrobial activity [59] but facilitates chromatin decondensation and the release of NETs. LL-37 contributes to nuclear membrane disruption and plays an active role in the formation and stabilization of NETs.

NETs coated with LL-37 are resistant to degradation by bacterial nuclease and may therefore be more effective in constraining microbial pathogens. While NETs are essential for defense due to their antimicrobial properties, excessive NET formation triggers a cascade of pathological tissue damage and severe inflammation [59,60], driven by the release of granular enzymes from neutrophils. The resulting release of additional inflammatory cytokines and cytotoxic debris [61] directly activates microglia and astrocytes, inducing the release of TNFα, IL-1β, and IL-6, which directly compromise the structural integrity of the blood–brain barrier (BBB) [62].

Due to their ability to rapidly extravasate and alter vascularization [63], neutrophils can cause collateral tissue damage [63,64].

NETosis acts as a double-edged mechanism that contributes to tissue damage and causes inflammation [30].

Notably, inhibitors of neutrophils and NETs have shown therapeutic strategies in AD [65].

### 3.2. The Role of Chloride Intracellular Channel 1 (CLIC1) in Neurodegeneration

Chloride Intracellular Channel 1 (CLIC1) was originally identified in monocytes [66,67] and can insert into cellular membranes from the aqueous phase [68]. In patients with neurodegeneration, CLIC1 tends to accumulate on the membrane of circulating monocytes, making it a potential indicator for early diagnosis [66]. LL-37 appears to act as a primary driver or key initiator of CLIC1 membrane translocation and activation in microglia. LL-37 directly binds to CLIC1 and promotes its transfer to the cell membrane, acting as an endogenous agonist that contributes to microglial hyperactivation, neuroinflammation, and neurotoxicity [30]. A recent study demonstrated that LL-37-mediated CLIC1 activation is a pivotal event in microglial pathogenic responses associated with AD.

Aβ also upregulates a CLIC1-mediated chloride current in microglia; current-clamp experiments have shown that Aβ alters the resting membrane potential of microglial cells through activation of NADPH oxidase (NOX2), causing depolarization [69,70], while activation of the CLIC1 current reduces depolarization [71]. Exposure of microglia to Aβ in vitro induces ROS generation through NADPH oxidase [69,72]. The membrane-associated NOX2 complex transfers electrons from intracellular NADPH to extracellular oxygen. The ROS generated through the CLIC1/NOX2 complex acts as a signaling molecule to activate the NLRP3 inflammasome, which leads to caspase-1 activation and the release of pro-inflammatory cytokines such as IL-1β and IL-18 [71].

At this stage, activated microglia releases additional toxic mediators including IL-1ß and TNF-α which promote the formation of amyloid fibrils and plaques. In the presence of Aβ and LL-37, CLIC 1 is no longer a harmless soluble protein, but becomes dangerous in AD [30]. However, pharmacological blockage of CLIC1 significantly inhibits the pathological effect induced by both Aβ and LL-37 and has a specific role in initiating and facilitating microglial ROS production, which may represent a promising novel therapeutic target to counteract neurodegeneration in AD [67].

### 3.3. Biological Properties of LL-37 and Aβ: Mechanisms of Interaction

LL-37 and Aβ are amphipathic α-helical peptides that insert into lipid bilayers, disrupt cellular membranes, and exhibit a natural propensity to self-aggregate. In the brain, LL-37 is upregulated under inflammatory conditions and can bind Aβ, thereby modulating its aggregation dynamics.

**Figure 3:**
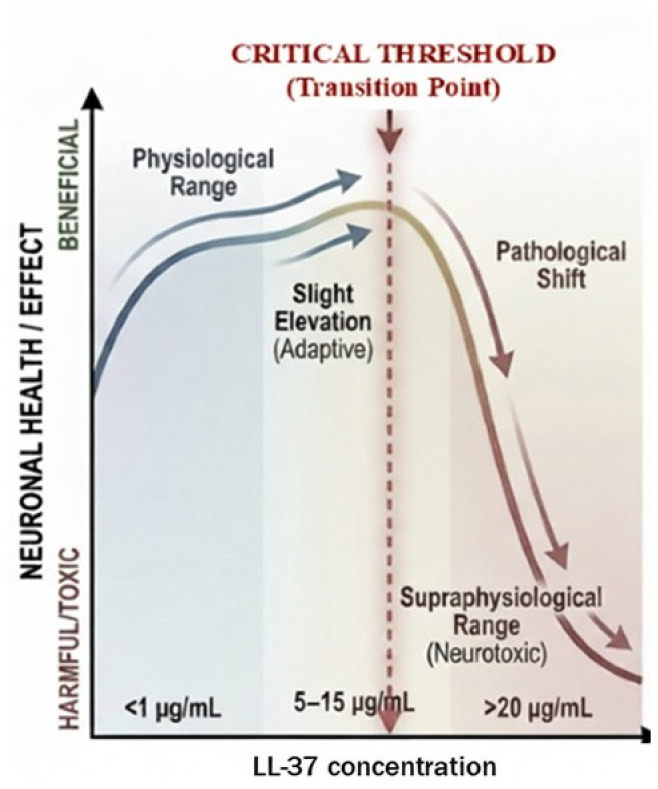
Supraphysiological concentration. Critical threshold where LL-37 transition from protective to neurotoxic.

Experimental studies [49] have demonstrated the inhibitory effect of LL-37 on Aβ oligomer fibril formation. LL-37 shows higher affinity for low-molecular-weight (MW) Aβ oligomers than for high-MW Aβ oligomers [49], suggesting a mechanism by which LL-37 prevents the formation of Aβ oligomers into fibrils. The interaction is driven by electrostatic attractions between the positively charged LL-37 and negatively charged Aβ_42_ oligomers, as well as hydrophobic interactions [73]. Structural comparison reveals extensive and striking parallels between Aβ and LL-37 (Table 1) [50]. Transmission electron microscopy has shown that LL-37 inhibits the formation of β-sheet-rich Aβ_1–42_ fibrils. Increased LL-37 expression in the brain correlates with reduced Aβ deposition [71,74]. Both peptides have protective roles as AMPs in the innate immune system but can transition into pathological drivers in AD when chronic inflammation or amyloidosis perturb their regulatory balance, leading to abnormal aggregation states driven by infections or chronic stress. This pathological shift is reflected in the cerebrospinal fluid (CSF) of AD patients where decreased Aβ levels and elevated LL-37 levels are often observed (Figure 3). However, under chronic inflammation conditions, LL-37 expression becomes dysregulated, leading to supraphysiological concentrations (Figure 3). Within the 5–20 µM range, LL-37 transitions from a protective molecule to a cytotoxic and neurotoxic one [65]. In such environments, LL-37 may instead promote the formation of amorphous aggregates or stabilize membrane-disrupting oligomers [57], shifting the balance toward cytotoxic outcomes [65].

Although both LL-37 and Aβ can independently induce neuroinflammation by activating intracellular proinflammatory pathways and promoting cytokine release, their interaction modifies this behavior. When the two peptides are present at equimolar concentrations, they show 90% less pro-inflammatory activity than either peptide alone. At sub-equimolar ratio, LL-37 only partially inhibits at a less than 1:1 molar ratio, only slowing but not fully preventing fibril formation. Only at a 1:1 molar ratio does LL-37 completely block the formation of Aβ_42_ fibrils [79,80].

## 4. The Role of Lipid Membranes and Detergents on Aβ and LL-37 Dynamics

Lipid membranes and micelles play a crucial role in modulating both Aβ and LL-37 by promoting aggregation through increased local concentration and altering biological activity, including enhanced toxicity for Aβ and increasing LL-37-mediated membrane disruption [81]. Elevated cholesterol levels in the plasma membrane increase Aβ generation and strongly correlate with AD progression. Interactions between cholesterol, gangliosides, and phospholipids facilitate Aβ aggregation at the membrane surface and can lead to membrane disruption, possibly through channel-like pore formation. This results in a perturbation of intracellular calcium homeostasis and suggests that Aβ–lipid interactions at the membrane may be responsible for the neurotoxic cascade in AD [82].

Lipids, fatty acids, and detergents accelerate fibrilization under various conditions [83]. A critical factor is the peptide-to-lipid or peptide-to-detergent ratio [84,85]. At low lipid concentrations, vesicles or micelles can confine peptides, increasing local concentration and driving aggregation through mass action [74]. At higher concentrations, aggregation is enhanced when the detergent:protein ratio falls below the number of detergent molecules required for micelle formation and is inhibited once each peptide is isolated in a separate micelle [79]. Diky et al. [84] stated that membrane interactions can induce conformational changes that favor intermolecular interactions, leading to aggregation; detergents at concentrations that favor aggregation appear to induce intermediate conformations of α-synuclein [84]. LL-37 induces positive curvature strain in lipid bilayers and also alters bilayer properties, such as hydrophobic thickness and area per lipid. In particular, Aβ oligomers display strong affinity for synaptic membranes and can induce synaptic dysfunction, a proposed primary cause of cognitive impairment in AD [76]. LL-37 undergoes a structural transition from an unstructured monomer in solution to an α-helical conformation at higher peptide concentrations or in the presence of lipid membranes. Cell membranes can act as catalysts of fibril formation as the local chemical environment reduces the free-energy barrier to peptide aggregation [79].

Aggregation of molecules often occurs at solubility limits. If a compound has amphiphilic properties, it preferentially aggregates between two media [19,86] where hydrophobic hydration is minimized. During micelle formation, van der Walls interactions between hydrophobic tails contribute to this process [19].

However, micelles are not a suitable membrane mimetic for studying AMPs as the absence of native-like lipid–peptide interactions with both the head groups and hydrophobic acyl chains limits their ability to reproduce self-assembling peptides and oligomer formations [87], thus limiting their use in experimental models.

LL-37 exists as monomers but can also oligomerize into dimers and tetramers when exposed to detergents under certain conditions [53].

## 5. Pathophysiology of Bacterial Lipopolysaccharide (LPS)

LPS does not cause AD or PD directly, but acts as a potent, common environmental catalyst that accelerates underlying proteinopathies through a shared neuroinflammatory pathway, making the gut–brain–microglia axis a crucial therapeutic target [88]. Interestingly, brain microbial load increases with age partly due to the age-related decline in Aβ clearance rate as well as other AD risk factors, such as increased blood pressure and cognitive decline. Chronic inflammation caused by toxic Aβ aggregates leads to the breakdown of the BBB, promoting microbial infiltration into the brain in AD [89].

LPS is a major component of the outer membrane of Gram-negative bacteria and is also known as endotoxin. Upon activation, LPS induces amyloid precursor protein (APP) expression and the release in monocytes of Aβ peptides, microglia, and astrocytes. Structurally, LPS displays surfactant properties (Figure 1B) due to its hydrophobic alkylic chain and anionic-phosphate headgroup, and can assemble into various physical structures such as micelles (Figure 1B) [21] or bilayers. When incorporated into Aβ micelles, LPS promotes heterogeneous nucleation on non-Aβ seeds which dominate the nucleation process, generating fibrils that are indistinguishable from those formed through classical micelle-mediated nucleation (Figure 1D). In this contest, LPS acts as a promoter of fibrillogenesis. The positive charge of antimicrobial peptides enhances their ability to bind to LPS [21]. Lipid A, a component of LPS, consists of a glucosamine disaccharide, two phosphates, and six acyl groups with long alkyl chains. The presence of these saturated fatty acids with 12–14 carbon chains reduce surface and interfacial tension, exhibiting a characteristic detergent-like behavior [90].

Long-term exposure to circulating endotoxin is not fully mediated by blood cytokines, but partly through activation [91,92] of TLR4-CD14/TLR2 receptors on microglia. LPS triggers NFkB-mediated increases in proinflammatory cytokines such as IL-6, TNF-α, and NOS2, which in turn promote Aβ production and accumulation [93] in AD. The failure of mucosal barriers transforms LPS from a localized structural component of gut and oral bacteria into a systemic threat. The resulting chronic inflammation triggers macrophages to release inflammatory cytokines that weaken the tight junctions of the BBB. Since Aβ_42_ is also an agonist for TLR4 receptors, this may create a pathological feedback loop contributing to AD progression [94]. LPS, the TLR4 receptor complex, and Gram-negative bacteria may be targets for treatment or the prevention of sporadic AD [95].

LPS stimulates microglia via TLR4 to produce nitric oxide (NO) and proinflammatory cytokines. When LPS is combined with IFNγ from infiltrating T cells, microglia and astrocytes express high levels of inducible nitric oxide-synthase (NOS). The resulting NO, when combined with either hypoxia or superoxide from NADPH oxidase, becomes neurotoxic [96].

LL-37 interacts with LPS, peptidoglycan, bacterial membranes, cytoplasmic proteins, and DNA, ultimately leading to microbial death [75,97].

In addition, LL-37 exerts indirect antibacterial effects by binding and neutralizing LPS (Figure 2B) and lipoteichoic acid (LTA), thereby dissolving bacterial biofilms [53].

### 5.1. Exposure to LPS Accelerates Neurodegeneration in AD

The mechanisms of AD and PD come together when LPS crosses the compromised BBB or the inflammatory signals propagate along the vagus nerve, binding microglia via the TLR4 receptor.

In AD, the burden of microorganisms in the brain increases with age. This rise is partly due to the age-related decline in Aβ clearance, hypertension, cognitive impairment, and the breakdown of the BBB caused by the accumulation of toxic Aβ aggregates [89] that are often associated with aging.

Elevated endotoxin levels have been reported to increase two to threefold higher in both the blood and brain of AD patients [93], and endotoxin has also been detected within amyloid plaques.

The causative agent of stomach ulcers, *Helicobacter pylori*, has also been suggested to be associated with AD serum. IgG and IgA antibodies against H. pylori occurred in a higher percentage in a group of AD patients [21]. The long-term effects of persistent or lifelong repeated infections may differ in different hosts, according to their general health, pharmacological treatments, and genetic or concurrent diseases [27].

Reduction in microbial dysbiosis has been shown to decrease plaque burden and microglial activation in an amyloid model of AD mice.

Under normal conditions, LPS does not cross intact BBB; its entry into the aging brain likely depends on cofactors such as ischemia, hypoxia, peripheral cytokines, and BBB-compromised areas.

Genetic factors further modulate the impact of endotoxin in AD. Carriers of APOE4—the strongest genetic factor for sporadic AD [75,77]—are more sensitive to LPS, showing higher Aβ levels after LPS exposure, and also have increased susceptibility to infection and cardiovascular disease. Variants in TLR4 [78] and its co-receptor TREM2 are also associated with increased AD risk, suggesting a genetic link between endotoxin signaling and AD pathogenesis [75,77].

People with periodontitis show elevated circulating blood endotoxin [98,99,100,101] and display a higher risk of AD and a faster cognitive decline, reinforcing the connection between LPS and AD pathogenesis. Refs. [21,27] discussed the possibility that microorganisms might participate in the formation of senile plaques, proposing a slow-acting infectious agent acquired early in life that becomes pathogenic decades later [17]. Aging is associated with a higher Gram-negative bacteria load in the gut, and antibiotic-induced perturbations in gut microbial diversity were shown to influence neuroinflammation and amyloid deposition in an AD mouse model [102]. Even if the peripheral source of LPS is completely eradicated in the gut microbiome, the misfolded proteins continue to fuel the microglial cytokine storm on their own.

### 5.2. LPS-Driven Neuroinflammation in Parkinson’s Disease (PD)

In Parkinson’s disease, α-synuclein (αS) aggregation originates in the gut and gastrointestinal dysfunction is one of the earliest non-motor symptoms of PD. [103]. Patients show increased intestinal permeability and elevated levels of LPS binding protein (LBP), reflecting higher circulation of endotoxin [104]. Elevated endotoxins stimulate macrophages to increase αS production and promote fibrillization. LPS acts as a potent catalyst that accelerates fibril deposition. This contributes to the degeneration of dopaminergic neurons in the substantia nigra, partly through activation of the NLRP3 inflammasome. Neuroinflammation is an essential protective response to pathogens, but chronic activation can lead to pathological states such as PD and other neurodegenerative disorders [104,105].

Proteins implicated in protein misfolding diseases, including αS, act as natural AMPs. Under infection-induced neuroinflammatory conditions, αS aggregation can increase facilitating neurodegeneration [105,106]. Once Aβ plaques (in AD) or αS Lewy bodies in PD reach critical mass, they become inflammatory triggers. They bind to the same microglial TLR4 receptors that LPS originally activated.

Dysbiosis further contributes to this process. For example, an overgrowth of *Enterobacteriaceae* elevates serum LPS and other microbial metabolites which cross the intestinal wall into the bloodstream. Dysbiosis can reduce the expression of tight-junction proteins such as occludin, increasing intestinal permeability. BBB breakdown contributes to the destruction of dopaminergic neurons in the substantia nigra, correlating with PD progression. Dopaminergic neurons cannot survive this oxidative stress, and the αS arriving from the gut or misfolding locally due to stress aggregates into toxic Lewy bodies.

The gut microbiome of PD patients differs from that of healthy controls and is associated with motor symptoms [107,108]. This suggests that alteration in the intestinal microbiota directly influences the course of disease, supporting the concept that gut-derived inflammation and endotoxin exposure contribute to neurodegeneration in PD. Common genetic variants also contribute to so-called ‘idiopathic’ PD, but the overall heritable component of disease is estimated to be around 35%,5 [107].

## 6. Engineered Amyloid Proteins with Antimicrobial Activity

Artificial amyloid materials offer a sustainable alternative strategy for biomedical application by utilizing the natural self-assembly mechanisms of peptides and proteins. Engineered peptide can be designed for use as a drug carrier and to improve drug targeting precision and release efficiency [109].

Engineered amyloid proteins are typically designed to maintain amphiphilic properties while removing native toxic segments. As a result, they do not aggregate pathologically or induce cytotoxicity, binding directly to amyloid oligomers and interfering with their ability to seed further plaque formation [110].

Amphiphilic antimicrobial peptide-based biomaterials have been designed by using Aβ_42_ as a template, and have been developed for use as drug delivery systems [111]. They can simultaneously inhibit toxic aggregation while maintaining strong antimicrobial activity against various pathogens.

Structural analogs of LL-3, such as kLL-39, have been engineered to exhibit enhanced antimicrobial activity and reduced cytotoxicity towards host cells [110].

Like classical AMPs, amyloid-derived peptide form ion channels or pores that cause cell lysis. Some can penetrate pathogens and induce the aggregation of essential intracellular proteins, leading to cell death. Engineered amyloids can bind selectively to microbial proteins, altering protein conformation and inhibiting their activity.

A recently designed amyloid-based peptide named Amy-Cat [111] consists of a short amyloid-forming segment and a cationic domain, forming a primary amphipathic structure. The Amy-Cat peptides exhibit strong bactericidal activity against Gram-negative and Gram-positive bacterial strains, with low minimum inhibitory and hemolytic concentrations. Synthetic amylin and Aβ have also been shown to enhance antibacterial responses, suggesting that cooperation between amyloid peptides can drive protective activity against neural infection [112,113].

Engineered amyloid peptides offer a promising platform for therapeutic development combining antimicrobial functions with the capacity to modulate pathological aggregation processes [114].

## 7. Conclusions

Antigenic stimulation may play a critical role in the development of different amyloid fibrils. Immunological imbalance processes emerge as host defense mechanisms that begin to deteriorate with age and comorbidities; however, the cytokine pattern observed in subjects affected by neurodegeneration is not simply an amplification of immune changes occurring with age. Aβ is produced to kill microbes, yet at high concentrations it becomes neurotoxic, damaging the neurons it was meant to protect. The process of Aβ fibrillogenesis strongly depends on initial peptide concentrations. Consequently, a distinct nucleation mechanism dominates in each different concentration domain. Aβ and LL-37 share striking biochemical similarities: comparable size and molecular weight, an amphipathic α-helical structure, and a tendency to self-aggregate. They are both host defense peptides and may be co-regulated as part of the innate immune response, capable of binding and interacting with each other during antimicrobial activity. LL-37 binds to Aβ_1–42_, inhibiting the formation of long fibrils but also potentially stabilizing smaller, more neurotoxic oligomers or hetero-oligomers that act as off-pathway aggregates, contributing to AD pathology. A potential disease-modifying approach may emerge from targeting the roots’ neuroinflammatory and amyloid-driven pathways through LL-37 modulation combined with vitamin D therapy. Vitamin D receptor (VDR) and retinoid X receptor (RXR) are implicated in AD and regulate LL-37 expression. Vitamin D3 treatment reduces cerebral Aβ accumulation and improves cognition in AD mouse models, while RXR activation reduced neuronal loss and enhanced cognitive performance in an aggressive AD mouse model. CLIC1 also represents a promising therapeutic target. Inhibitors that prevent CLIC membrane insertion such as the peptide CLIC1pep have shown the ability to block pathological phenotypes triggered by both LL-37 and Aβ. These agents may therefore represent a novel strategy to counteract neurodegeneration. Despite significant progress, the molecular mechanism underlying amyloid-driven inflammation and infection linked to neurodegeneration remains incompletely understood. Further research is essential to deepen understanding of these pathways and to develop targeted therapeutic strategies capable of modifying disease progression.

## Figures and Tables

**Table 1:** Structural and Functional Comparison of Aβ and LL-37.

Feature	Amyloid-Beta (Aβ)	LL-37	References
Origin	Cleavage product of APP (Aβ1-40/Aβ1-42)	Cleaved from hCAP 18 (only human cathelicidin)	[3,75]
Physiological role	Innate immunity, synaptic regulation, antimicrobial peptide	Broad spectrum AMP, immunomodulator, wound healing	[6,8,52]
Structure	Amphipathic peptide, prone to β-sheet transitions	Amphipathic α-helical peptide	[10,11,53]
Aggregation tendency	oligomers, protofibrils, fibrils, plaques	oligomers, dimers, tetramers	[14,76]
Mechanism of action	Inserts into lipid bilayers, forms pores, disrupts Ca^2+^ homeostasis	Inserts into lipid bilayers, disrupts pathogens via pore formation	[26,46]
Antimicrobial activity	Moderate; acts like an AMP during infection	Strong, broad-spectrum antimicrobial activity	[6,7,9]
Role in neuroinflammation	Activates microglia via TLR4 → IL 1β, TNF α	Induces TNF α, IL 6; activates NLRP3; synergizes with Aβ	[29,30]
Interaction between Aβ and LL-37	LL 37 binds Aβ1 42 → inhibits fibrils but stabilizes oligomers	Binds Aβ oligomers → prevents fibrils but may form toxic hetero-oligomers	[49,50]
Pathological role	Central in AD pathology, plaque formation, synaptic toxicity	Dysregulated expression contributes to neuroinflammation and microglial activation	[22,30]
Genetic links	APP, PSEN1/2; APOE4 increases Aβ load	Expression regulated by VDR/RXR; influenced by vitamin D levels	[49,77,78]

## Data Availability

No new data were created or analyzed in this study. Data sharing is not applicable to this article.
